# Patterns of Recurrence After Liver Transplantation for Nonresectable Liver Metastases from Colorectal Cancer

**DOI:** 10.1245/s10434-013-3449-9

**Published:** 2013-12-27

**Authors:** Morten Hagness, Aksel Foss, Tor Skatvedt Egge, Svein Dueland

**Affiliations:** 1 Section for Transplantation Surgery, Department of Transplantation Medicine, Oslo University Hospital, Oslo, Norway; 2 Department of Radiology and Nuclear Medicine, Oslo University Hospital, Oslo, Norway; 3 Department of Oncology, Oslo University Hospital, Oslo, Norway; 4 Oslo University Hospital, Institute of Clinical Medicine, University of Oslo, Oslo, Norway

## Abstract

**Background:**

Surgical resection is the only curative modality for colorectal liver metastases (CLM), and the pattern of recurrences after resection affects survival. In a prospective study of liver transplantation (Lt) for nonresectable CLM we have shown a 5-year overall survival rate of 60 %, but 19 of 21 experienced recurrence. This study reports the pattern of recurrences after Lt for CLM and the effect on survival.

**Methods:**

Characterization of metastatic lesions in a prospective study for Lt for nonresectable CLM was performed (*n* = 21). The study included reexamination of chest computed tomographic scans taken before Lt.

**Results:**

At the time of first recurrence, 16 were a single site, and three were multiple sites. Thirteen of the single sites were pulmonary recurrences. The pulmonary recurrences appeared early and were slow growing, and several were accessible to surgical treatment. When chest computed tomographic scans were reexamined, seven patients had pulmonary nodules at the time of Lt without an effect on survival. There was no first single-site hepatic recurrence. Six of the seven patients who developed metastases to the transplanted liver died from metastatic disease.

**Conclusions:**

The pulmonary recurrences after Lt for CLM were of an indolent character, even those that were present at the time of Lt. This contrasts with the finding of metastases to the transplanted liver, which was prognostically adverse. The lack of single hepatic first-site recurrences and hepatic metastases only as part of disseminated disease is different from the pattern of recurrence after liver resection. This suggests two distinct mechanisms for hepatic recurrences after resection for CLM.

Chemotherapy as the sole treatment of colorectal liver metastases (CLM) is palliative only, and the 5-year overall survival (OS) after the start of first-line chemotherapy is approximately 10 %.^1^ Surgical treatment of CLM is potentially curative, and the median 5-year OS is 38 %, ranging from 16 to 74 %.^2^ Recurrence after liver resection for CLM happens in 60–70 % of the patients.^3^–^5^ The first site of recurrence is most frequently liver only (28–45 %), followed by lung only (17–27 %), multiple sites (28–30 %), and locoregional or other single sites (9–12 %).^3^,^4^,^6^–^8^ Recently there have been several reports on the effect of the pattern of the first site of recurrence on outcome after liver resection for CLM. Not surprisingly, the survival is better for single-site recurrences than for multiple sites.^6^,^9^ In a recent report from Memorial Sloan-Kettering Cancer Center (MSKCC), the best outcome after single-site lung metastases was demonstrated, and survival after single-site hepatic recurrences was placed in between that after pulmonary and multiple-site recurrences.^6^ Other reports show no difference in survival between lung and liver recurrences.^7^,^9^

CLM is currently regarded as a contraindication for liver transplantation (Lt). However, in a prospective study on Lt for nonresectable CLM (*n* = 21), we showed a 5-year OS of 60 % (95 % confidence interval, 34–85 %).^10^ Nineteen of the 21 patients experienced recurrence of disease. A significant proportion of the recurrences were accessible for surgery, and at last follow-up, 33 % of the patients had no evidence of disease.^10^

The primary aim of the present study was to describe the pattern of recurrences after Lt for CLM and to explore the effect of these patterns on survival. Patterns of recurrence after Lt for CLM have, to our knowledge, never been described before. Also, complete removal of the affected liver may give novel information about the biology of metastatic spread, because it excludes the mechanism of relapse caused by residual tumor cells situated in the liver. Because of frequent pulmonary relapses, another aim of the study was to reassess chest computed tomographic (CT) scans on patients with pulmonary recurrence, to pinpoint the timing of appearance.

## Methods

### Patient Selection

A total of 21 patients with nonresectable CLM underwent Lt in an open prospective pilot study; main inclusion criteria were nonresectable CLM without signs of extrahepatic disease and a minimum of 6 weeks of chemotherapy.^10^ The absence of extrahepatic disease was assessed by chest, abdominal, and pelvic CT scans and whole-body positron emission tomography/CT scan. The examinations as part of the pretransplantation procedure were done at various referring hospitals or at the transplantation center as part of their routine diagnostic work. CT scans taken at other hospitals were reexamined at our department of radiology. The thicknesses of slices were 2.5–3 mm. If no sign of extrahepatic malignancy was found, the patient was put on a waiting list for Lt. At admission for Lt, a repeat chest CT scan was performed and assessed by the radiologist on call at the transplantation center. All of these were described as negative regarding pulmonary metastasis. A staging laparotomy was performed at the time of Lt. All chest CT scans taken before Lt and during follow-up were retrospectively reassessed by an experienced radiologist (T.S.E.) as part of the present study.

The immunosuppression protocol consisted of sirolimus, mycophenolate mofetil, corticosteroids, and induction with basiliximab. Sirolimus was introduced on the first postoperative day, aiming for a trough level of 5–10 ng/ml during the first 4 weeks and 10–20 ng/ml thereafter. Corticosteroids were tapered to 0 or 5 mg daily at 1–2 months after Lt. No adjuvant chemotherapy was given.

After discharge, chest, abdominal, and pelvic CT scans were performed every 3 months for the first year and thereafter every 6 months according to study protocol. Patients with relapse of malignant disease were treated individually.

The study was approved by the ethical and institutional review board (S-05409 Regional Ethics Committee).

### Statistical Analysis

Data were continuously registered in case report forms. Survival data were estimated by using the Kaplan–Meier method. Log-rank tests were used to compare survival between subgroups. For all tests, a two-sided *p* < 0.05 was considered statistically significant. Analyses were performed with SigmaPlot for Windows Version 11.0.

## Results

### Diagnosis of Recurrence

Time to recurrence was defined as time to metastasis or recurrence of the primary cancer during the follow-up period. All hepatic recurrences were diagnosed by the characteristic appearance of new lesions in the transplanted liver on CT scans and/or ultrasound. In one patient, resection of the metastatic lesion in the transplanted liver was performed, and histology of the lesion showed adenocarcinoma. In another patient, fine-needle aspiration cytology of the lesion verified the diagnosis of metastases from colorectal adenocarcinoma. In two other patients, stereotactic radiation toward the liver was performed. In one of these patients there was a transient decrease in carcinoembryonic antigen from 112 to 49 μg/l.

For pulmonary relapses, the time to recurrence was defined as when the pulmonary deposit was first described as certain metastases on chest CT scans. In one case, the hepatic recurrence was diagnosed on an additional abdominal CT scan taken on clinical indications 40 days before planned chest CT follow-up. This examination also revealed pulmonary recurrence in the lower part of the lungs. Accordingly, this patient was registered as having simultaneous liver and lung relapse. The relapses were diagnosed by CT examinations in 17 cases, the recurrent rectal cancers were identified by magnetic resonance examination, and one of the lymph node metastases was diagnosed by positron emission tomography/CT scan.

### Patients, Extent of Cancer Disease, and Treatment

The baseline demographics and clinical outcome of the 21 patients who underwent transplantation in the pilot study were described previously.^10^ Briefly, the median time of follow-up was 27 months (range, 8–60 months). The Kaplan-Meier estimate for OS was 95, 68, and 60 % at 1, 3, and 5 years, respectively. The Kaplan–Meier estimate for disease-free survival was 35 and 0 % at 1 and 3 years, respectively. Two grafts were lost because of hepatic artery thrombosis, and the patients underwent retransplantation at 4 and 12 days after first Lt.

The inclusion criteria were quite wide regarding the characteristics of the primary or hepatic tumor, previous treatment, and response to chemotherapy. No limits were given to characteristics such as carcinoembryonic antigen levels, synchronous disease, or extent of hepatic tumor load. Accordingly, the study population was heterogeneous regarding characteristics of colorectal cancer disease and previous treatments. Fourteen of the 21 patients had progressive disease on chemotherapy at the time of Lt, and these patients had inferior survival.^10^ The metastatic tumor load in the liver was extensive (Table 1).

**Table 1:** Baseline characteristics for patients undergoing liver transplantation for nonresectable colorectal liver metastases

Characteristic	Value
Age at treatment, years, median (range)	56 (45–65)
Sex, *n* (%)	
Male	13 (62 %)
Female	8 (38 %)
Cancer treatment before transplantation, *n* (%)	
Liver resection/RFA	6 (28 %)
Chemotherapy	
Lines of chemotherapy received before Lt, *n* (%)	
1 line	9 (43 %)
2 or 3 lines	12 (57 %)
Progression on chemotherapy at Lt, *n* (%)	14 (66 %)
Tumor characteristics, liver metastases	
Number of metastases, median (range)^a^	8 (4–40)
Diameter of largest metastasis, cm, median (range)^a^	4.5 (2.8–13.0)
Carcinoembryonic antigen levels at time of Lt, median (range)	15 (1–2002)

*Lt* liver transplantation, *RFA* radiofrequency ablation

^a^Largest number/diameter measured at CT scans before Lt or examination of explanted liver

### Pattern of Recurrence

A total of 19 of the 21 patients developed recurrences. All patients observed for more than 11 months experienced recurrence of disease. The median time to recurrence was 6 months (range 2–24 months). At the end of follow-up, seven patients were alive with no evidence of disease, eight patients were alive with recurrence, and six patients were deceased. The observational time for patients with no evidence of disease was a median of 11 months (range, 6.5–25 months) since transplantation or last resection. Time from diagnosis of recurrence to surgical treatment was 7.9 months (range 0–21 months) in five patients. No patient died of causes other than relapse of the malignant disease.

**Fig. 1:**
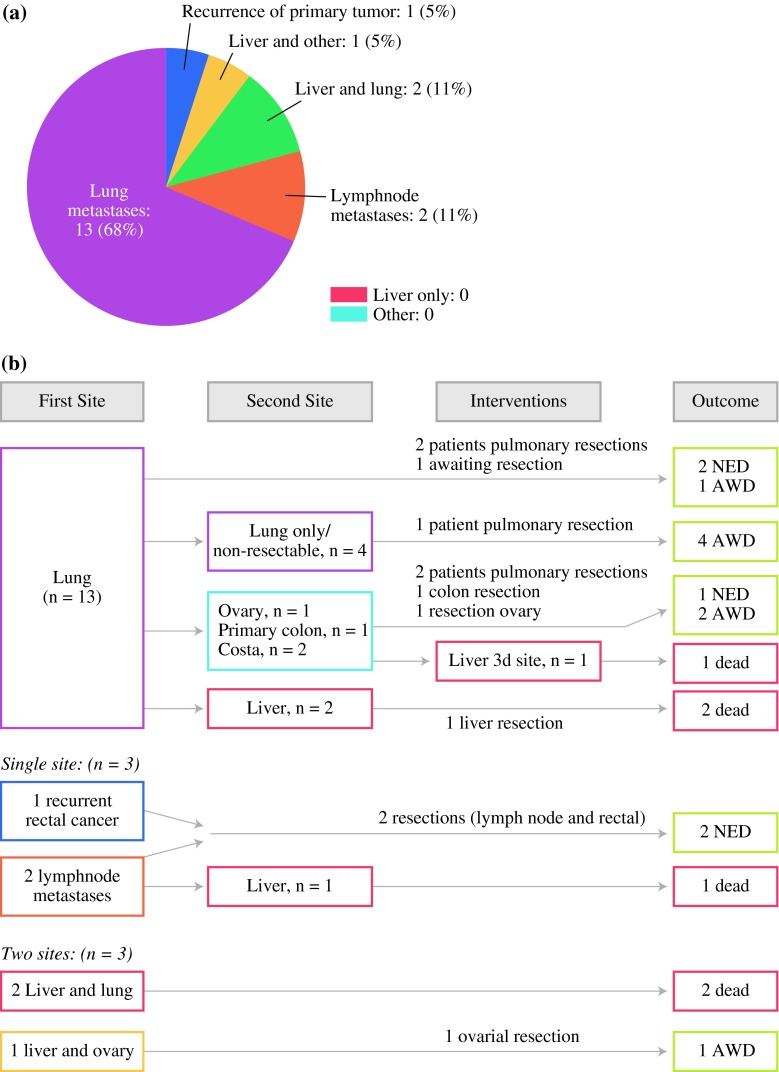
**a** Distribution of first-site recurrences after liver transplantation (Lt) for colorectal cancer for nonresectable liver metastases. Nineteen patients had experienced recurrence at the end of follow-up. **b** Surgical interventions and outcome according to distribution of first and second site of recurrence after Lt. Of the 21 patients included in study, six were dead, all because of disseminated cancer disease; seven were with no evidence of disease (NED); and the remaining eight were alive with disease (AWD). No patient was lost to follow-up

The initial recurrence patterns were distributed as shown in Fig. 1a: 68 % lung metastases, 11 % liver and lung metastases, 11 % lymph node metastases, and 5 % liver and ovarian metastasis. Five percent experienced local recurrence of a rectal tumor as the first recurrence. No patient had liver-only metastasis as the first site. The median time from Lt to initial recurrence was 4 months (range 2–24 months) for lung only, 6 months (range 2–15 months) for multiple site recurrences, and 12 months (range 5–14 months) for other single-site recurrences. Sixty-two percent of the patients with single-site lung metastasis experienced recurrence of disease within the first year.

The lung was the first single site of recurrence in 13 patients (Fig. 1b). Seven of these patients did not develop other metastatic deposits, and three of these seven underwent pulmonary resections. At the end of follow-up, all seven patients were alive, two of them with no evidence of disease at 9.5 and 25.0 months after resection. In this group, one patient had a solitary very slowly growing pulmonary tumor which had been observed for 23 months without reaching 1 cm in diameter. Four of the patients with lung as the first site of recurrence experienced other locations as second sites; two patients had port-site metastases to the costae after thoracoscopic pulmonary resection, one patient was later diagnosed with a new primary colon cancer, and one patient had ovarian metastasis. The new primary colon cancer was resected, and the patient had no evidence of disease 13.1 months after pulmonary resection at the end of follow-up. In two cases, the liver was the second site of tumor recurrence, and one of the patients with port-site metastasis to the costae experienced liver as the third metastatic site 30 months after Lt. All three patients died from metastatic disease.

There were three single-site recurrences other than pulmonary after Lt. One was a recurrent rectal cancer, and two patients experienced metastases to paraaortal lymph nodes. The patient with local relapse of rectal cancer received chemoradiation and subsequent surgery of the rectal relapse, and this patient had no evidence of disease 18.4 months after surgery at the end of follow-up. One of the patients with lymph node metastasis developed liver metastasis as a second-site recurrence and died. The other underwent resection, received postoperative radiation (50 Gy), and had no evidence of disease 6.4 months after radiation treatment at the end of follow-up (Fig. 1b).

Three patients had recurrences at two sites simultaneously. The liver was involved in all three. Two of these patients died at 6 and 26 months after Lt. One was alive with disease after an ovarian resection for metastasis, but with metastases in the liver and lungs.

### Survival after Site of Recurrence

**Fig. 2:**
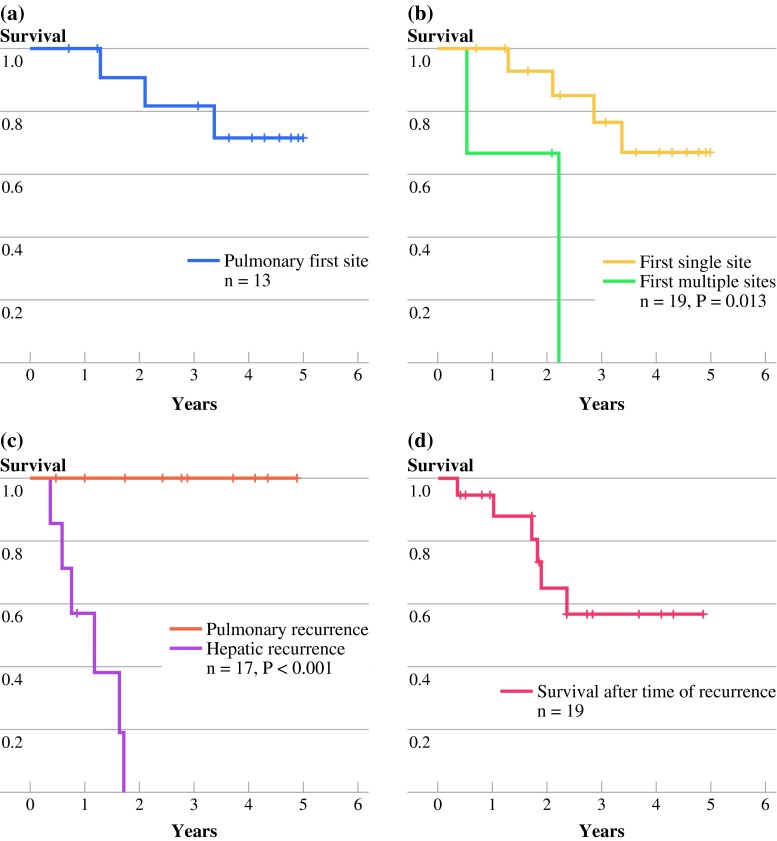
Nineteen of 21 patients who experienced relapse after liver transplantation for colorectal liver metastasis were assessed. All deaths were due to disseminated cancer disease. **a** Thirteen patients had pulmonary-first metastases, and the 5-year survival from the time of Lt was 72 % (95 % confidence interval 44–99 %). **b** Three patients had two metastatic sites (two patients had liver and lung, and one patient had liver and ovary) as first sites (*green line*), whereas the remaining 16 patients had single sites (pulmonary or lymph node metastases or recurrent rectal cancer; *yellow line*). The survival plot shows time from Lt. **c** Time from diagnosis of hepatic metastases to death (*purple line*). The *orange line* represents patients who experienced pulmonary metastases but no hepatic relapse. From the diagnosis of pulmonary metastases to the end of follow-up, there were no deaths in this group. **d** Overall survival from time of recurrence. The log-rank test was used for calculation of *p*-values in panels (**b**, **c**)

Patients with a pulmonary first-site recurrence had a 5-year survival of 72 % (95 % confidence interval 44–99 %; Fig. 2a) from the time of Lt. The 16 patients who had single-site recurrences, both lung and other, had significantly better survival than the three with multiple-site recurrences, of whom all had liver metastases (*p* = 0.013; Fig. 2b)

**Fig. 3:**
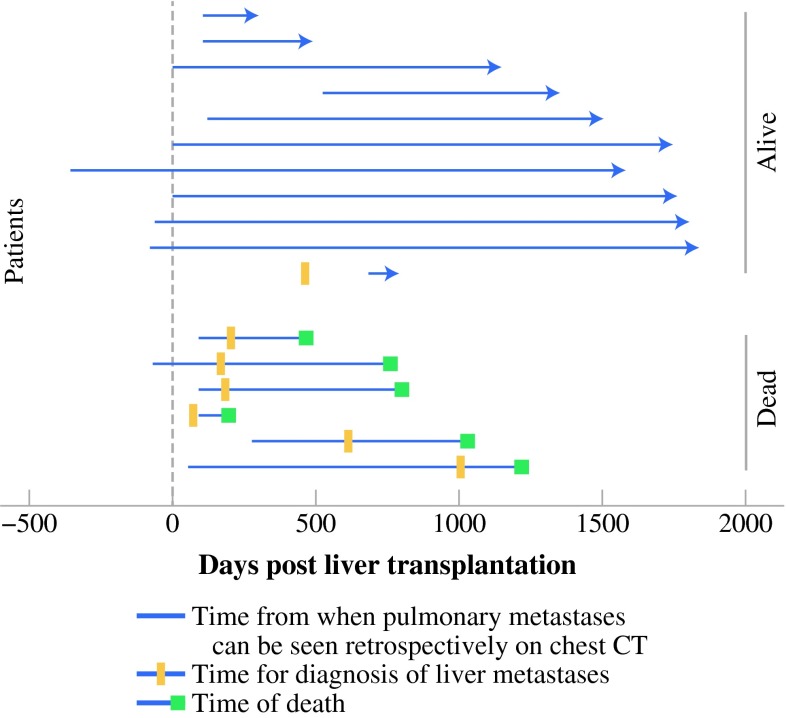
Previous chest CT scans on 17 patients who experienced lung metastases were reassessed. Tracing back from obvious metastatic manifestations, prior metastatic nodules were found in several patients. In seven patients they were present at or even before liver transplantation. The plot shows the course of individual patients. Each *line* is the time from the earliest manifestation of metastases seen retrospectively on CT, the *vertical line* at point 0 is the time of liver transplantation, and the *red bar* is the time of diagnosis of hepatic metastases. The patients alive are in *upper part* of the plot, and the dead, below

Seven patients developed hepatic metastases. The median time from Lt to liver metastases was 6 months (range 2–30 months). One of these patients underwent hepatic resection, two received stereotactic radiation toward the liver, and subsequently one of these two patients underwent transcatheter arterial chemoembolization treatment. At the end of follow-up, six of these seven patients were dead. The median time from diagnosis of liver metastases to death was 14 months (range 4–21 months; Figs. 2c, 3). In contrast, all 12 patients with recurrences that did not include the liver were alive at the end of follow-up. Patients with pulmonary metastases and no liver metastases (ten patients) had a significantly better survival from the time of relapse compared with patients diagnosed with hepatic recurrence (*p* < 0.001; Fig. 2c). The 5-year OS after the diagnosis of recurrence was 57 % (Fig. 2d).

Tracing back from evident metastases on CT scans, seven patients had pulmonary metastases at time of Lt. Four of them had metastases 2–12 months before Lt. The presence of metastases at the time of Lt did not have a negative effect on survival. Only one of these patients died during follow-up, and six of them were among the ones with the longest survival in the study (Fig. 3). In some cases, CT scans showed nodules with the same size and appearance as the future evident metastases, but without further growth, they were retrospectively considered benign.

## Discussion

Seven of 21 patients experienced hepatic metastases after Lt. The finding of hepatic recurrences of colorectal cancer in a transplanted liver has, to our knowledge, not been described previously. All patients in the study had excised primary tumor, and all patients had undergone strict preoperative screening with CT and positron emission tomography/CT scans and colonoscopy. No single-site hepatic first recurrence was observed in the present study. This contrasts with what is seen after liver resection for CLM, where the proportion of hepatic first-site recurrences is high (31–45 %).^4^,^6^,^7^ In a study of patients who underwent hepatic and pulmonary resections, as many as 70 % had hepatic first-site recurrences.^11^ Further, the prognosis after first-site liver metastases described in literature is at the same level as or slightly worse than that for pulmonary metastases, whereas in our study the emergence of liver metastases was seen only as part of disseminated disease and was a devastating prognostic sign. The median time from diagnosis of liver metastases to death was 14 months (range 4–21 months). This is in line with multiple-site recurrences after liver resection of CLM reported from MSKCC, where the median OS from the time of recurrence was 13 months (range 11–16 months), compared with 24 months (range 17–30 months) in patients with single-site liver recurrences.^6^ All six patients who died in our study had hepatic metastases, and six of the seven patients who experienced hepatic metastases were dead at the end of follow-up.

Regarding metastatic spread of colorectal cancer, the transplanted liver is situated downstream from the lung. Following primary colorectal cancer, the portal flow distributes more than 90 % of the metastases to the liver, whereas the incidence of lung metastases without liver involvement is less than 10 % and appears from both colon and rectal cancer.^12^ Metastases downstream of the lung without lung involvement after primary colorectal cancer is exceedingly rare.^13^ Further, metastases downstream of the lung are associated with larger pulmonary tumors, and these findings underscore the filtering function of the lungs.^12^ Downstream metastases such as skeletal metastases after primary colorectal cancer are later events than lung metastases and exhibit a poor prognosis. This provides a potential explanation for why metastases to the transplanted liver were seen only as part of disseminated disease and associated with an adverse prognosis. Further, the unfortunate prognosis of nonresectable hepatic metastases and hepatic recurrence in new liver in contrast to the comparatively benign features of pulmonary metastases underline the importance of hepatic disease control for survival. This is in line with results after locally directed modalities such as hepatic artery infusion after conversion of nonresectable CLM to resection.^14^

The lack of first-site liver recurrence and the finding that neohepatic metastases appeared only as part of disseminated disease suggest that there might be two distinct mechanisms for hepatic recurrences after liver resection of CLM. Metastases to the transplanted liver were of extrahepatic origin, whereas the liver-only first-site recurrences that are seen most frequently in liver resection studies are, at least partly, caused by residual tumor cells in the liver. Although these cells have been lodged through portal flow and developed through the metastatic cascade at an earlier point, the current mechanism would probably be more dependent on chemokines and other factors of homing.^15^–^17^

The overall number of patients who developed pulmonary metastases in the study was extensive (*n* = 17). This is probably due to vast hepatic tumor load, unfortunate characteristics of the primary tumor, and the fact that 29 % of patients had progressed on all standard lines of chemotherapy at Lt.^10^ Further, the time interval from Lt to initial recurrence of lung metastases was short (the median time was 4 months), and 62 % of the patients with lung-first recurrence experienced recurrence within the first year. This contrasts with findings after hepatic resection in which lung recurrences appeared later. In one study, the median time was 16 months, and in the MSKCC study, only 28 % of the lung-first metastases recurred during the first year.^4^,^6^ In the current study, most of the pulmonary metastases were slow growing, and several were accessible to surgery. Patients with pulmonary first-site recurrences had a 5-year OS of 72 % (95 % confidence interval 44–99 %). Further, no patient with pulmonary metastases alone or in combination with extrahepatic recurrences was deceased at the end of follow-up. These are superior to results in CLM resection studies. In a recent study, the 5-year OS was approximately 40 % from the time of initial pulmonary recurrence after hepatic resection for CLM.^6^ In addition, in our study, pulmonary metastases were present at the time of Lt, or even before Lt in seven patients. Our initial fear was accelerated growth of overlooked metastases when patients were given the continuous immunosuppressive medication. However, six of the seven patients who actually had pulmonary metastases at the time of Lt were among those who have survived longest in the study. This pilot study cannot give possible explanations for the slow growth rate of pulmonary metastases. One can speculate whether the antineoplastic properties of mammalian target of rapamycin inhibitors or the removal of hepatic tumor load which would otherwise have killed the patients and thus set the stage for more slowly growing pulmonary metastases contributed to this phenomenon. The standard chemotherapy given before Lt may have suppressed the growth of manifest pulmonary metastases preoperatively. Thus, the short disease-free survival caused by increased growth of lung metastases might be a consequence of termination of chemotherapy at Lt. Nevertheless, a 5-year OS of 60 % demonstrates that the immunosuppressive treatment used in the study did not induce accelerated growth of pulmonary malignancy.

The finding of pulmonary metastases at the time of Lt and previously by reexamination of CT scans illustrates limitations in the preoperative screening procedure. Indeterminate pulmonary nodules are quite common on pulmonary CT studies. It is known from preoperative screening of CLM that some nodules represent early metastases.^18^,^19^ In a recent report from MSKCC, 43 % had subcentimeter pulmonary nodules at the time of liver resection for CLM, and 35 % of these were proven to be metastatic disease. There was a trend toward shorter progression-free survival in patients with subcentimeter pulmonary nodules compared with those without.^18^ In this study, the nodules were small, uncharacteristic, and drawn attention to only retrospectively. Several other small nodules of identical appearance present on the CT examinations did not progress into metastases.

Conclusively, after Lt for nonresectable CLM, pulmonary metastases appeared frequently and early but were relatively indolent. This contrasts with the course of the patients who developed hepatic metastases to the transplanted liver. These appeared exclusively as part of disseminated disease, and there were no first-site liver-only recurrences. The presence of lung metastases at the time of Lt did not affect survival negatively.
